# In defense of diversity statements in medical education

**DOI:** 10.1093/jlb/lsag010

**Published:** 2026-03-26

**Authors:** Undaleeb Dairkee, Gregory Curfman, Carmel Shachar

**Affiliations:** Health Law and Policy Clinic, Harvard Law School, 1585 Massachusetts Avenue, Cambridge, MA 02138, USA; Journal of the American Medical Association (JAMA), 330 N. Wabash Avenue, Chicago, Il 60611, USA; Health Law and Policy Clinic, Harvard Law School, 1585 Massachusetts Avenue, Cambridge, MA 02138, USA

## I. INTRODUCTION

The decision in Students *for Fair Admission v. President & Fellows of Harvard College* (*SFFA*),^1^ and a May 9, 2025, memorandum^2^ from Secretary of Defense Pete Hegseth prohibiting the use of race as an admission consideration for military service academies, have hindered the ability of the nation’s universities to foster racial and ethnic diversity. We support the premise that it is important for universities, and in particular medical schools, to promote diversity through other means. One strategy used by universities is the requirement that applicants for professorships, professors seeking promotion or tenure, and in some cases, students applying for admission, must submit diversity statements as part of the application process. The statements generally include information about what the applicant has previously done to promote diversity, equity, and inclusion (DEI), and what the applicant would plan to do if hired, promoted, or accepted for admission by the university.

Universities often provide explicit directions, in the form of rubrics, for how to write a successful diversity statement and specific language to use or avoid. The University of California, Berkeley, has a sample rubric, which departments are encouraged to use and modify for their needs. This rubric includes themes that assess a candidate’s previous history and future plans to promote diversity within their classroom environment. Candidates are scored based on the following criteria: prior teaching experience and teaching approaches that highlight efforts to remove barriers and support the success and participation of all students through an inclusive curriculum; teaching interests and plans that foster an inclusive research environment that removes barriers and promotes equitable access; examples of efforts by the candidate to remove barriers, support the success of students and other academics, increase the participation of those from groups underrepresented in higher education; and previous examples of and future plans to create inclusive climates.^3^ Princeton University, which places importance on promoting diversity among its students and faculty, defines diversity statements (though the University refers to them as perspective statements) as an outline of how an applicant will advance an institution’s approach to fostering an inclusive learning environment.^4^ As the reader may notice, neither example focuses on a candidates’ race or ethnicity as a factor in developing a strong diversity statement. Rather, the focus is on inclusivity and fostering a learning environment that encourages students to assess problems using diverse perspectives and create solutions that are responsive to varied needs.

Diversity statements with these characteristics are helpful in hiring medical school faculty that will better prepare medical students to understand the diverse patient populations they will serve as physicians. We focus on medical schools because of the important effect medical education has on shaping the ability of future physicians to deliver culturally competent care and, therefore, address health disparities. The ability to recognize and respect cultural differences, a key component of cultural competency, enhances relationships and promotes inclusivity. This improves the doctor-patient relationship, addresses health disparities, and leads to better health outcomes.

Medical schools can play an integral role in developing culturally competent physicians by pursuing faculty who can mentor all students to provide sensitive, culturally competent care. The ability to apply that knowledge compassionately is strongly correlated with having empathy for the patient, which can be enhanced by understanding the cultural, racial, and socioeconomic backgrounds of the patient. One effective mechanism for teaching empathy is to ensure that medical faculty are well versed in the norms of different cultures and are understanding of the generational toll of discrimination, racism, and bias in health care. Additionally, it is critical that faculty recognize the adverse effect of economic hardships on the health of people of all racial and ethnic backgrounds.

Developing a physician workforce that is able to respond to patients of a variety of backgrounds does not necessarily require that the students or faculty come from diverse backgrounds. It does require, however, that students are taught by faculty, regardless of their race or ethnicity, that understand the importance of treating patients in a culturally appropriate manner. Faculty mentors who can effectively teach the delivery of culturally competent care may be of any race or ethnicity but must have a commitment to addressing these concerns in their pedagogy.

Medical schools have a compelling justification to incorporate diversity statements into their faculty hiring and promotion to develop a profession that can adequately respond to patient needs. This article explores the case law shaping the use of diversity statements in higher education. It then discusses exceptions to *SFFA’s* prohibition on race-conscious policies in admissions to suggest an opportunity for medical schools to incorporate diversity statements into their faculty hiring and promotions. It also articulates why medical schools, as compared to more general undergraduate institutions, have a heightened interest in incorporating diversity initiatives, even if the prevailing political winds blow hard against them.

## II. A CHALLENGING MOMENT FOR DEI

Political winds blow against DEI, most notably with the Trump Administration’s executive order^5^ seeking to end DEI practices in higher education and to ensure that all institutions of higher education that receive federal funding follow *SFFA*. The National Institutes of Health has recently stated that grants will not be made to research institutions that operate programs with the purpose of advancing or promoting DEI.^6^ Predating the second Trump Administration, the Supreme Court’s decision in *SFFA*^7^ has made it considerably more difficult to achieve a racially and ethnically diverse physician workforce. Following the Court’s decision in *Regents of the University of California v. Bakke*^4^ nearly 50 years ago, subsequently affirmed in *Grutter v. Bollinger*,^5^ universities and their medical schools were able to use race as a plus factor in admissions. In those cases, the Court ruled that holistic affirmative action programs aimed at achieving racial and ethnic diversity in university classrooms were a compelling state interest and were narrowly tailored, sufficient to pass strict scrutiny, the most rigorous standard of review.^8^ In *SFFA*, the Court changed course and ruled that ‘race-conscious admissions’ are unconstitutional under the Equal Protection Clause of the Fourteenth Amendment. The Court noted that any race-conscious policies would have to survive strict scrutiny, meaning that the racial classification must be used to further a compelling governmental interest and be narrowly tailored to achieve that interest.^9^

Other scholars have noted that *SFFA* is confusingly vague on what it means to pursue race-neutral admissions policies,^10^ meaning that universities are now trying to retool their admissions programs with relatively little guidance. Universities, such as the *SFFA* defendants Harvard and University of North Carolina, have interpreted *SFFA* to mean that they may not consider the race or ethnic background of applicants in making decisions about admissions.^11^ President Trump, in his March 4, 2025, address to Congress, lauded *SFFA* as ‘brave and powerful’.^12^

Furthermore, in light of *SFFA*, universities have become wary of using diversity initiatives elsewhere, including faculty hiring. While this article was in preparation, the Harvard Faculty of Arts and Sciences, Massachusetts Institute of Technology (MIT), and the University of Michigan announced that they would no longer be using diversity statements in faculty hiring.^13^ Harvard administrators received strong negative feedback from faculty about the statements, and the MIT president noted that the statements amounted to compelled speech and did not work. That three prominent universities have nearly simultaneously eliminated diversity statements in faculty hiring may have wider ramifications.^14^ Other universities may well follow suit, and six states have passed laws eliminating the use of diversity statements by their public universities.^15^

Some of this caution is likely driven by the current political landscape. One of President Trump’s first actions on reentering the White House was to issue an executive order prohibiting the use of DEI initiatives in higher education.^16^ A notable focus in this executive order was requiring compliance with *SFFA* for all institutions of higher learning that receive federal funding. In the Trump Administration’s battles with universities, DEI hiring programs have been flagged as problematic by Government leadership. For example, in its April 11, 2025, letter, the Trump Administration demanded that Harvard only engage in ‘merit-based hiring’ and ‘cease all preferences based on race, color, religion, sex, or national origin throughout its hiring, promotion, compensation, and related practices among faculty, staff, and leadership’.^17^

It is also clear that the Trump Administration considers *SFFA* applicable not just to admissions but also to other university business. In his address to Congress, President Trump framed *SFFA* as preventing hiring professors on anything but merit.^18^ The Office of Civil Rights of the Department of Education recently issued a letter stating that institutions receiving federal financial support may not consider race in hiring and promotion.^19^ In a related development, the National Institutes of Health has indicated that grants will not be awarded to research institutions having DEI programs.^20^

Many universities will have to weigh not just whether their hiring practices are constitutional, but if they can withstand the political pressures from the Administration to avoid engaging in such practices. This article does not scrutinize the Administration’s actions, which may or may not stand the tests of litigation and time, but rather focuses on whether diversity statements used for hiring in medical schools can be constitutional. We flag the political risks of utilizing diversity statements for faculty hiring and promotion to reflect on the understandable trepidation that many universities have about incorporating required DEI initiatives.

## III. MANDATED DIVERSITY STATEMENTS CAN BE CONSTITUTIONAL

The rationale for the use of mandated diversity statements may be viewed from the perspective of both policy and law. There are at least three attributes of diversity statements that support their use on policy grounds. The universal rationale of diversity statements for faculty hiring, promotion, and tenure are intended to create a more inclusive university community.^21^ The presence of an inclusive teaching faculty at a university may also attract a more diverse pool of student applicants, which *pari passu* will result in a more diverse student population without necessarily relying on affirmative action in admissions. More specific to the medical education context, mandated diversity statements highlighting the importance of diversity and inclusivity as a guiding principle of the institution are well positioned to have spillover effects on other aspects of university decision-making. In medical schools, this includes the hiring and promotion of faculty who may not themselves come from racially diverse backgrounds but understand and support the importance of teaching medicine in a way that recognizes racial and cultural differences in patients and their perception of medicine. It is important to acknowledge, however, that despite the logic underpinning the use of mandated diversity statements, there is little empirical evidence that they have a causal effect on the diversity or inclusiveness of the faculty or student body.

Mandated diversity statements can be challenged and have been critiqued on the grounds that they may be performative. As candidates are told by the university what a successful statement consists of, applicants may simply construct a statement to fit the purpose. There may sometimes be a risk of the candidate using the statement as a signal of their own virtuous motives, but which may not be sincere. Or perhaps, in some cases, the authors may use them as proxies for their own race or ethnicity, and universities may likewise utilize diversity statements to identify a candidate’s race or ethnicity.

## IV. FREE SPEECH ANALYSIS

As a matter of law, the requirement by universities for diversity statements is complex and controversial.^22^ Some legal scholars have pointed to two aspects of constitutional law, both involving the First Amendment free speech clause, that challenge the legal stance of diversity statements.^23^ The First Amendment often forbids discrimination based on the content of speech, and the Amendment universally forbids discrimination based on viewpoint.^24^ Diversity statements are content-based, since the university specifies the message that is at the core of the statement. Diversity statements are also generally regarded as viewpoint-based because most suspect that universities will generally accept only one perspective on the value of diversity in university life (ie, that it is important).^25^ While these considerations undoubtedly apply to public universities, which are an extension of the government to which the First Amendment inevitably applies, many private universities also voluntarily commit to following the speech principles of the First Amendment in developing university policies. However, in contrast to public universities, private universities are not legally required to adhere to First Amendment principles.

Some observers have claimed that diversity statement requirements for faculty hiring or promotion have the potential to run counter to the First Amendment based on either content or viewpoint discrimination.^26^ However, it is not immediately evident that diversity statements would necessarily qualify as a form of content discrimination since universities are free to request statements from applicants on any subject that the university believes is relevant to its academic and pedagogical mission. The issue of viewpoint discrimination, however, requires a closer analysis. The constitutional touchstone regarding viewpoint discrimination in universities was stated cogently by the Supreme Court in *Keyishian v. Board of Regents*:

Our Nation is deeply committed to safeguarding academic freedom, which is of transcendent value to all of us, and not merely to the teachers concerned. That freedom is therefore a special concern of the First Amendment, which does not tolerate laws that cast a pall of orthodoxy over the classroom.^27^

In hiring professors, universities routinely examine professors’ viewpoints about the subject matter of their research and teaching. Consider, for example, two neurology professors applying for a position at a medical school, who have different views on the neurological regulation of memory. The medical school may surely consider the differing viewpoints of the professors in making the hiring selection of that professor the school believes is most tenable.

In the case of diversity statements, some legal scholars have argued that they are a fundamentally different matter.^28^ They are required of all applicants, and they do not address specific areas of scholarship, but instead deal with more general matters that some scholars refer to as political or ideological.^29^ The university requires a particular point of view, and opposing points of view are not acceptable. This moves closer to qualifying as viewpoint discrimination. Still, given that universities may normalize the requirement for diversity statements, arguendo diversity statements may then become immune from critique. Universities may contend that diversity statements reflect the learning mission and adhere to accreditation requirements.

A legal case arguing that mandated diversity statements are a form of viewpoint discrimination will be subject to heightened (or intermediate) scrutiny,^30^ such as in the case of *Haltigan v. Drake*, discussed subsequently.^31^ In order to survive heightened scrutiny, a university utilizing diversity statements would have to convince the court that there was a compelling interest in their goals and that diversity statements are an appropriately tailored solution to accomplish those goals. Therefore, it is not out of the question for the use of diversity statements to pass this level of scrutiny, but it does depend on whether the defendant could argue for a compelling interest and justify diversity statements as sufficiently narrowly tailored.^32^

While we caution readers not to conflate the case law governing university admissions policies—which is based in Fourteenth Amendment jurisprudence—with the law governing faculty diversity statements—which as discussed previously is primarily First Amendment jurisprudence—we believe that *SFFA*^33^ and its related cases can be helpful in constructing a defense of the use of diversity statements in faculty hiring and promotion. A compelling interest is a compelling interest, whether the Fourteenth or First Amendment imposes heightened scrutiny. Because most of the key compelling speech cases do not arise in the university context, *SFFA* may provide the best insights into what a compelling justification for diversity conscious policies in education might look like.

When it comes to the use of race based criteria, the public interpretation of the Supreme Court’s recent jurisprudence seems to be that *SFFA* ended all raced-based affirmative action.^34^ A closer reading, however, indicates that although the Court struck down Harvard’s and University of North Carolina’s race conscious admission policies, there are nevertheless some opportunities to continue these practices, at least from a constitutional law perspective. In particular, the jurisprudence that governs admissions is related but distinct from the case law governing required faculty statements. Furthermore, even when *SFFA* may have implications for faculty statements, there is a line of reasoning developing that certain types of schools may have heightened interests in diversity that could justify DEI initiatives. The justices did not foreclose the use of race entirely.^35^ Particularly relevant for medical schools is Chief Justice Roberts’ treatment of military academies under the ‘distinct interests’ rationale. Roberts seemed to imply that *SFFA* is not intended to apply to these institutions, flagging that ‘[t]his opinion also does not address the issue, in light of the potentially distinct interests that military academies may present’.^36^ Justice Sotomayor, in her dissent, located Roberts’ distinction between military academies and civilian universities in ‘national security interests’ and then argued that this distinction is not warranted.^37^

Almost immediately, Roberts’ limited discussion of military academies was tested in court when Students for Fair Admissions brought lawsuits challenging the US Naval Academy’s^38^ and the US Military Academy at West Point’s^39^ race-based admission policies. In both cases, the military academy in question was able at least to suggest to the court at hand that it had a compelling interest in promoting officer corps diversity through narrowly tailored race-conscious admissions policies. In the Naval Academy’s case, the judge, after a nine-day bench trial, ruled that the Naval Academy could continue to use race in its admission process. The judge agreed there is a compelling national security interest in having a diverse officer corps. The judge in the Naval Academy case relied significantly on Roberts’ Footnote 4 touching on military academies noting, ‘[i]n declining to apply Harvard to military service academies, however, the Court made clear that service academies may identify distinct and compelling governmental interests that justify their use of race in admissions’.^40^ Similarly, the judge in the West Point case harkened back to Roberts’ *SFFA* footnote to distinguish between the treatment of Harvard’s admissions policies and West Point’s. In rejecting SFFA’s demand for a preliminary injunction, the judge noted, ‘what was not compelling to the Supreme Court as regards civilian universities may in fact be compelling when raised in the context of West Point and national security interests. Indeed, these possibilities are precisely what *Harvard* itself left open, by declining to address the issue altogether, “in light of the *potentially* distinct interests that military academies *may* present.”’^41^ These cases are now settled because both the Naval Academy and West Point have ceased to consider race as a factor in admissions.^42^

A second constitutional question raised by mandated diversity statements is whether they represent a form of compelled speech, which is disfavored by First Amendment jurisprudence.^43^ While the First Amendment prohibits the government from passing laws infringing the speaker’s right to speak, First Amendment doctrine also prohibits laws that compel people to speak.^44^ The state cannot require individuals to deliver a message on behalf of the state.^45^ A recent Supreme Court case that addressed the constitutionality of compelled speech was *303 Creative v. Elenis*.^46^ This case involved a web designer who wanted to expand her business to create websites for weddings, but she did not want to create websites for same-sex weddings, stating that they were against her religious beliefs. The Court ruled in the designer’s favor, deciding that requiring her to create sites for same-sex weddings against her beliefs was a form of compelled speech in violation of the First Amendment. In this case, the Court judged that the state’s interest in compelling the designer to speak did not meet a standard of heightened scrutiny.^47^ Another recent Supreme Court case involving compelled speech was *National Institute for Family and Life Advocates v. Becerra*, a case involving a California law requiring crisis pregnancy centers, which are anti-abortion organizations offering other pregnancy services, to provide women visiting the centers with information about abortion and contraceptive options.^48^ The Supreme Court struck down the law as a violation of the First Amendment, concluding that the law compelled content-based speech and did not pass even intermediate-level scrutiny.^49^ A third recent compelled-speech case, *RJ Reynolds Tobacco Company v. Food and Drug Administration*,^50^ involved an FDA regulation that tobacco manufacturers must place graphic warning labels on all cigarette packages and advertising, for the purpose of informing consumers about the health hazards of cigarette smoking. The US Court of Appeals for the Fifth Circuit ruled that the mandated warning labels were a form of compelled commercial speech that was subject to a lower standard of review than compelled political speech, referred to as *Zauderer* review,^51^ and that the warning labels passed this standard of review. Taken together, these three cases demonstrate that courts may reach differing conclusions about the constitutionality of government-compelled speech depending on the form and context of the speech.^52^ While compelled political speech infrequently passes First Amendment review, compelled commercial speech may do so to provide information to the public (here the interest of the listener takes precedence over the interest of the speaker).^53^

## V. EXISTING CASE LAW ON DIVERSITY STATEMENTS

With these cases as background, how have courts addressed the constitutionality of diversity statements mandated by universities as a form of compelled speech?^54^ To date, the cases that have been brought have not reached a decision on the merits. One recent legal case, *Haltigan v. Drake*, challenged the constitutionality of university diversity statements.^55^ John Haltigan was interested in a psychology professorship at the University of California at Santa Cruz, but he declined to submit the diversity statement required by the University because he strongly opposed the use of DEI rubrics in the academy.^56^ He subsequently brought a lawsuit against the University of California in the US District Court for the Northern District of California claiming infringement of his First Amendment rights (*Haltigan v. Drake*). As he had not actually been denied a professorial position because he refused to submit a diversity statement, the court dismissed the case due to lack of standing. Haltigan then resubmitted a revised complaint. Haltigan, represented by the Pacific Legal Foundation, claimed that the mandated diversity statement, which he believes is tantamount to a loyalty oath, litmus test, and mandated orthodoxy, is a form of unconstitutional compelled speech and viewpoint discrimination.^57^

The *Haltigan* case is not ultimately instructive on whether mandatory diversity statements could survive heightened scrutiny. Because Haltigan did not ultimately apply for the position, the court determined that he had no concrete and particularized injury in fact and dismissed the case. But this resolution would not be available in a case in which a candidate did submit an application or a professor was required to produce a diversity statement to receive tenure. In another, similar case, *Palsgaard v. Christian*, the Foundation for Individual Rights and Expression (FIRE) filed suit against the California Community Colleges system for implementing a DEI mandate for evaluating professors.^58^ In response, the Community College system withdrew the DEI mandate, and the case was dismissed.^59^ As in *Haltigan*, this case was not decided on the merits. *Haltigan* and *Palsgaard* therefore bring us back to our original question, which is whether mandatory diversity statements used specifically by medical schools could survive a constitutional law challenge.

Considering that we are in an era in which diversity initiatives are scrutinized, it may be a close question for courts to as to whether mandated diversity statements as part of faculty hiring and promotion are compelled speech. If a plaintiff claimed that diversity statements represent a form of compelled speech, the legal standard of review would be heightened scrutiny, and as with viewpoint discrimination discussed previously, it is reasonable to believe that diversity statements as a form of compelled speech could satisfy this standard, which would require that diversity statements further an important government interest and to do so by means that are substantially related to that interest. The willingness and ability of professors to teach students to be comfortable with a wide variety of backgrounds is an essential part of the medical school pedagogical process, as discussed below, and may therefore qualify as a compelling state interest. As we will discuss and document in more detail subsequently, the need for a diverse physician workforce is an important priority for the health of the nation. We will make the case that achieving such a workforce (which we do not currently have) is a compelling interest of the state. The use of diversity statements as a means of accomplishing that goal, we will argue, is substantially related to that important public health interest.

## VI. MEDICAL SCHOOLS HAVE A COMPELLING INTEREST IN DIVERSITY

Jonathan Feingold draws from *SFFA* Footnote 4 that, ‘For institutions of higher education, Footnote 4 should invite the following question: Do we possess potentially distinct interests in diversity that SFFA did not address? If the answer is “yes,” that suggests a plausible path to defend a race-based admissions process’.^60^ This litmus test is one of the ways to demonstrate a compelling interest in diversity initiatives, whether they are admissions policies or faculty hiring requirements. Feingold indeed suggests that professional schools, including law and medical schools, are different from undergraduate research universities such as the plaintiffs in *SFFA*.^61^ This is because professional schools, unlike undergraduate research universities, have a specific commitment to educate and develop a workforce that will serve the health and welfare of the broader community. To better develop this argument that they possess a distinct interest in diversity, medical schools should carefully study the arguments made by the Naval Academy and West Point in their admissions cases to build the argument that there is a compelling interest in developing a medical faculty that values diversity, equity, and inclusion. Medical schools are different than undergraduate institutions in that they are professional schools, with the unique responsibility of shaping the practice of medicine, and therefore the health of the country, well into the future. This means they are not established solely for the noble goal of advancing human understanding, even when it comes to biomedical research. Rather, medical schools have a primary purpose of training the next generation of the physician workforce. A medical school that advances scientific knowledge but does not graduate physicians to treat patients would not be considered a medical school at all. And a medical school that turned out physicians who provided substandard care to patients would be considered flawed.

Because medical schools are the gatekeepers to the medical profession, they have a responsibility to American society to improve medical care for all Americans. This includes ensuring minority communities, who historically see the greatest health disparities, receive care that addresses their needs in a compassionate and culturally appropriate manner. Requiring diversity statements in the hiring and promotion process for medical faculty helps address both current health disparities that exist and past harms that still affect the way some populations view healthcare providers and the care they recommend. Of course, an analogous argument can be made for other parts of the academy and other professional pursuits. Our purpose in this article is to focus on the medical profession, but we do not argue that the medical profession is alone in the need for a racially and ethnically inclusive workforce.

Poor health outcomes driven by health disparities are a serious concern in the USA. Health outcomes in the USA are worse than those of other high-income countries yet, as a nation, we spend more on health care than those countries.^62^ A Commonwealth Fund study that ranked health care system performance across ten high-income countries found that the USA stood number ten in overall ranking, access to care, and health outcomes, and number nine in administrative efficiency and equity.^63^

The unequal distribution of poor health outcomes among US patients suggests that disparities drive some of the poor performance in the American health care system. For example, in obstetrics a Black mother is up to four times more likely than a White mother to die from childbirth-related complications-with significant disparities existing even when controlling for socio-economic status, lifestyle, insurance coverage, and other factors.^64^ This can be partially explained by care that is biased given that a survey conducted by the Kaiser Family Foundation in 2023 found that ‘22% of Black women who have been pregnant or gave birth in the past ten years say they were refused pain medication they thought they needed’.^65^ In contrast, Black providers are more likely than others to accurately assess Black patients’ pain tolerance and prescribe the correct amount of pain medication as a result.^66^ If providers are not listening to their patients when they ask for pain medication while in labor, what other signs and symptoms are these physicians missing that a more culturally sensitive provider would have noticed?

The pattern of patients of color, particularly Black patients, having better outcomes with physicians from similar backgrounds holds true in other specialties. In pediatrics, having a Black physician more than doubles the likelihood that a high-risk Black newborn will live.^67^ In pediatric cardiology, Black and Hispanic children with heart conditions are more likely to die than their White counterparts, in part potentially due to in-hospital differences in care.^68^ These are examples of how outcomes can be impacted, both positively and negatively, in part based on whether the care a patient receives is sensitive to their needs.

Beyond the ethical imperative to address health disparities, there is a compelling state interest to narrow or eliminate health disparities from an economic perspective. The financial toll posed by health inequities is substantial. A study conducted by Deloitte found that ‘health inequities account for approximately $320 billion in annual health care spending’, and if inequities are left unchecked, they could result in annual health care spending of $1 trillion or more by 2040.^69^

Research has demonstrated that when provider care teams are diverse or when providers are exposed to physicians with an understanding of the impact of racially and culturally sensitive care, they have a different approach to their medical practice that can lead to improved health outcomes for their patients of color. For example, communities with more Black physicians have a strong correlation with positive health outcomes for Black members of those communities. One study led by the Health Resources and Services Administration found that ‘every 10% increase in the representation of Black primary care physicians in a county was associated with 30.6 days of greater life expectancy among Black people in that county, regardless of whether a Black doctor treated those patients’.^70^ To be clear, the takeaway from this body of research should not be that only Black physicians can successfully treat Black patients. Instead, it is that the medical profession must include physician leaders that can push providers from overlooking or neglecting certain patients’ needs. By doing so, physicians can build trust in their patients, which is the foundation of a sound physician-patient relationship.^71^

Medical schools are key institutions in building a culturally competent physician workforce and one of sufficient size to meet the healthcare needs of our society. By 2050 approximately 50 per cent of the US population will consist of people of color.^72^ This increase underscores the importance of providing patients with care that is individualized and considers their diverse backgrounds and particular needs. Statistics show, however, that the US physician workforce falls short of mirroring the society that it serves. Data for 2022 show that only 5.2 per cent of US physicians are Black, and only 6.3 per cent are Hispanic.^73^ Unfortunately, in part because of *SFFA,* medical schools have struggled to increase the diversity of their incoming classes. In 2024, the number of Black matriculants to medical schools declined by 11.6 per cent from the previous year, despite the number of Black applicants slightly increasing by 2.8 per cent between 2023 and 2024. Similarly, Hispanic applicants increased by 2.2 per cent from 2023 to 2024, but Hispanic matriculants decreased during this time period by 10.8 per cent. American Indian or Native American matriculants declined by 22.1 per cent during this time.^74^

If medical schools are unable to mirror, within their student population, the diverse populations that require medical care, another solution must be sought. One such solution is to ensure that American medical schools are producing culturally competent graduates who understand the complexities involved in caring for diverse populations that extends beyond knowledge about disease processes and encompasses being taught the tenets of culturally competent care. Such care is defined as ‘care that respects diversity and cultural factors that can affect health and health care, such as language, communication styles, beliefs, attitudes, and behaviors’.^75^ There are biases that most of us inherently have that prevent us from fully appreciating other points of view. In the medical context, this can prove harmful for patients who are dismissed or are not fully heard by their providers, potentially leading to poor treatment adherence and, at times, harmful consequences.

The goal, therefore, in using diversity statements in hiring and promotion of medical school faculty is not necessarily having a more diverse faculty, but rather having a faculty that understands the effect that culturally appropriate care can have on health disparities that disproportionately affect communities of color and lesser means. To maintain accreditation, medical school curriculums must include cultural competency training including ‘the knowledge, skills, and core professional attributes needed to provide effective care in a multidimensional and diverse society’.^76^ This descriptive language is central to being a culturally competent physician. While accreditation standards for medical schools could change in the future, this core requirement would be increasingly difficult for medical schools to comply with if they are not hiring and promoting faculty who are well-versed in this area of pedagogy. Losing this competency is a real concern, which could occur if diversity statements are no longer permitted, given that the statements are a key method to facilitate hiring of competent people in this field.^77^

Medical schools, both through their basic science curricula and clinical training, help shape the medical profession. This includes ensuring communities of color, who historically see the greatest health disparities, receive care that addresses their needs in a compassionate and appropriate manner. Requiring diversity statements in the hiring and promotion of medical school faculty helps ensure that faculty are equipped to address the roots of health inequities.

## VII. CONCLUSION

Medical schools that require, or are considering requiring, diversity statements for faculty hiring and tenure decisions may be reasonably confident that they will not face legal repercussions. Principles of academic freedom favor the right of universities and their medical schools to determine the grounds for hiring and promoting professors, and mandated diversity statements fit securely into the criteria that universities may utilize as a matter of policy. Medical schools must also educate a physician workforce of sufficient magnitude to meet society’s healthcare needs. Given the foundational importance of achieving racial and ethnic diversity among medical school faculties and students as a compelling state interest, mandated diversity statements for medical schools appear to be on reasonably solid legal ground, and medical schools may employ diversity statements with the conviction that the statements are an acceptable constitutional strategy for achieving diversity among their faculty. Diversity statements represent good policy and good law.^78^ Medical schools must continue to promote racial and ethnic diversity as a way of broadening the nation’s physician workforce, which is among the highest priorities in medical education. A diverse physician workforce will inevitably contribute to building trustful relationships between doctors and patients, a critical dimension of high-quality healthcare.

## Footnotes

1 Students for Fair Admissions v. President and Fellows of Harvard College, 600 US 181 (2023).

2 Secretary of Defense Pete Hegseth, Memorandum for Senior Pentagon Leadership Defense Agency and DOD Field Activity Directors, May 9, 2025, Certification of Merit-Based Military Service Academy Admissions (accessed Oct. 12, 2025).

3
*Creating a Rubric to Assess Faculty Candidates*, UC Berkeley Office for Faculty Equity & Welfare, Creating a Rubric to Assess Faculty Candidates | Office for Faculty Equity & Welfare (accessed Oct. 12, 2025).

4
*Perspective Statements*, Princeton Center for Career Development, https://careerdevelopment.princeton.edu/guides/resume-cv-cover-letter-diversity-statement/diversity-statements (accessed Oct. 12, 2025).

5
*Ending Illegal Discrimination and Restoring Merit-Based Opportunity*, The White House (2025), https://www.whitehouse.gov/presidential-actions/2025/01/ending-illegal-discrimination-and-restoring-merit-based-opportunity/ (accessed Mar. 7, 2025).

6 Anil Oza, *NIH Bans All Future Grants to Universities with DEI Programs or Israel Boycotts*, STAT, Apr. 21, 2025, https://www.statnews.com/2025/04/21/trump-nih-grants-draft-rule-would-prohibit-university-dei-programs-israel-boycott/ (accessed Apr. 29, 2025).

7 SFFA v. Harvard, *supra* note 1.

8 Gregory Curfman, *Bakke Redux—Affirmative Action and Physician Diversity in Peril*, 50 Journal of Law, Medicine & Ethics 619 (2022).

9 SFFA v. Harvard, *supra* note 1, at 206.

10 Issa Kohler-Hausmann, *What Did SFFA Ban? Acting on the Basis of Race and Treating People as Equals*, 66 Ariz. L. Rev. 305 (2024).

11 Cam Kettles & Claire Yuan, *After Affirmative Action Falls, Students, Counselors, and Schools Seek New Roadmap for Admissions* | News | The Harvard Crimson, The Harvard Crimson, Aug. 22, 2023, https://www.thecrimson.com/article/2023/8/22/affirmative-action-application-process/ (accessed Mar. 18, 2025).

12
*Transcript of President Donald Trump’s speech to a joint session of Congress*, AP News (2025), https://apnews.com/article/trump-speech-congress-transcript-751b5891a3265ff1e5c1409c391fef7c (accessed Apr. 21, 2025).

13 Hartocollis Anemona, *MIT Will No Longer Require Diversity Statements for Hiring Faculty—The New York Times*, New York Times, May 6, 2024, https://www.nytimes.com/2024/05/06/us/mit-diversity-statements-faculty-hiring.html (accessed Apr. 22, 2025); Jessica Willis & Daniel Ortner, *MIT Stops Using DEI Statements in Faculty Hiring | The Foundation for Individual Rights and Expression*, FIRE (May 9, 2024), https://www.thefire.org/news/mit-stops-using-dei-statements-faculty-hiring (accessed Apr. 22, 2025); Colleen Mastony, *University Sets Policy on Diversity Statements*, The University Record, https://record.umich.edu/articles/university-sets-policy-on-diversity-statements/ (accessed Apr. 22, 2025); Tilly Robinson & Neil Shah, *Harvard Faculty of Arts and Sciences Will No Longer Require Diversity Statements | News*, The Harvard Crimson, https://www.thecrimson.com/article/2024/6/4/dei-faculty-hiring-stopped/ (accessed Apr. 22, 2025).

14 Willis and Ortner, *supra* note 16.

15 Johanna Alonso, *DEI Bans Flourished in 2024. Politicians Aren’t Finished*, Inside Higher Ed, https://www.insidehighered.com/news/faculty-issues/diversity-equity/2024/12/16/how-battle-over-dei-shook-out-2024-and-whats-come (accessed Apr. 22, 2025).

16 Ending Illegal Discrimination and Restoring Merit-Based Opportunity, *supra* note 8.

17 Pavan Thakkar, *Harvard Will Fight Trump’s Demands*, The Harvard Crimson, Apr. 15, 2025, https://www.thecrimson.com/article/2025/4/15/harvard-denies-trump-demands/ (accessed Apr. 23, 2025).

18 Transcript of President Donald Trump’s speech to a joint session of Congress, *supra* note 15.

19 Office for Civil Rights, United States Department of Education, Dear Colleague Letter, (2025), https://www.ed.gov/media/document/dear-colleague-letter-sffa-v-harvard-109506.pdf (accessed Apr. 21, 2025).

20 Oza, *supra* note 9.

21 Daniel Ortner, *In the Name of Diversity: Why Mandatory Diversity Statements Violate the First Amendment and Reduce Intellectual Diversity in Academia*, 70 Cath. U. L. Rev. 515 (2021).

22 FIRE Statement on the Use of Diversity, Equity, and Inclusion Criteria in Faculty Hiring and Evaluation | The Foundation for Individual Rights and Expression, https://www.thefire.org/research-learn/fire-statement-use-diversity-equity-and-inclusion-criteria-faculty-hiring-and# (accessed Apr. 21, 2025).

23 Brian Soucek, *Diversity Statements*, 55 UC Davis L. Rev. 1989 (2021).

24 Judith Areen, *Government as Educator: A New Understanding of First Amendment Protection of Academic Freedom and Governance*, 97 Georgetown Law Journal 945 (2009).

25 Ortner, *supra* note 24; Soucek, *supra* note 26.

26 Ortner, *supra* note 24.

27 Keyishian v. Board of Regents, 385 US 598 (1967).

28 Richard A. Epstein, *The Civil Rights Juggernaut David C. Baum Memorial Lecture on Civil Liberties and Civil Rights*, 2020 U. Ill. L. Rev. 1541 (2020); Brian Leiter, *The Legal Problem With Diversity Statements*, The Chronicle of Higher Education (2020), https://www.chronicle.com/article/the-legal-problem-with-diversity-statements/ (accessed Mar. 18, 2025).

29 Leiter, *supra* note 31.

30 Soucek, *supra* note 26.

31 Haltigan v. Drake, No. 23-cv-02437-EJD, 2024 WL 150729 (N.D. Cal. Jan. 12, 2024).

32
*Id.*

33 SFFA v. Harvard, *supra* note 1.

34 Leiter, *supra* note 31.

35 Jonathan P. Feingold, *Affirmative Action after SFFA JCUL Special Issue: What’s next? Diversity in Higher Education after SFFA v. Harvard/UNC*, 48 J.C. & U.L. 239 (2023); Jeffrey S. Lehman, *Don’t Misread SFFA v. Harvard, Inside Higher Ed*, https://www.insidehighered.com/opinion/views/2023/07/17/dont-misread-sffa-v-harvard-opinion (accessed Mar. 18, 2025).

36 SFFA, *supra* note 1.

37
*Id*. Sotomayor (dissenting) at 355.

38 Students for Fair Admissions v. United States Naval Academy, F.Supp.3d (2024) (S.D.N.Y.).

39 Students for Fair Admissions v United States Military Academy at West Point, 709 F.Supp.3d 118 (2024) (D. Md.).

40 Students for Fair Admissions v. United States Naval Academy, *supra* note 7, at 55.

41
*Id.* at 135 (quoting SFFA, 600 U.S. at 213 n.4).

42 Anemona Hartocollis, *Naval Academy Seeks Dismissal of Lawsuit After Dropping Race-Conscious Admissions*, New York Times, June 16, 2025. https://www.nytimes.com/2025/06/16/us/naval-academy-race-admissions-lawsuit.html (accessed Oct. 5, 2025).

43 David S. Han, *Compelled Speech and Doctrinal Fluidity Symposium: Compelled Speech: The Cutting Edge of First Amendment Jurisprudence*, 97 Ind. L.J. 841 (2022).

44 Eugene Volokh, *The Law of Compelled Speech Essay*, 97 Tex. L. Rev. 355 (2018); Vikram David Amar & Alan Brownstein, *Toward a More Explicit, Independent, Consistent and Nuanced Compelled Speech Doctrine*, 2020 U. Ill. L. Rev. 1 (2020).

45 Volokh, *supra* note 48.

46 303 Creative v. Elenis, 600 US 570 (US 2023).

47 Chad Flanders, *Compelling Interests and Compelled Speech The Evolution of Religious Liberty*, 70 Wayne L. Rev. 25 (2024).

48 National Institute of Family and Life Advocates v. Becerra, US 755 (US 2017).

49 Robert Post, *NIFLA and the Construction of Compelled Speech Doctrine* the Addison C. Harris Lecture, 97 Ind. L.J. 1071 (2022); Flanders, *supra* note 51.

50 RJ Reynolds Tobacco Company v. Food and Drug Administration, 96 F. 4th 863 (United States Court of Appeals, Fifth Circuit 2024).

51 Micah L. Berman, *Clarifying Standards for Compelled Commercial Speech Toward a Healthy First Amendment*, 50 Wash. U. J. L. & Pol’y 53 (2016); Clay Calvert, *Wither Zauderer, Blossom Heightened Scrutiny: How the Supreme Court’s 2018 Rulings in Becerra and Janus Exacerbate Problems with Compelled-Speech Jurisprudence*, 76 Wash. & Lee L. Rev. 1395 (2019).

52 Post, *supra* note 53.

53 Berman, *supra* note 55.

54 Erica Goldberg, *‘Good Orthodoxy’ and the Legacy of Barnette*, 13 FIU L. Rev. (2019), https://ecollections.law.fiu.edu/lawreview/vol13/iss4/6 (accessed Dec. 1, 2024).

55 Haltigan v. Drake, No. 23-cv-02437-EJD, 2024 WL 150729 (N.D. Cal. Jan. 12, 2024).

56
*Id.*

57 Fighting unconstitutional DEI ‘loyalty oaths’ at the University of California, Pacific Legal Foundation, https://pacificlegal.org/case/haltigan-uc-diversity-statements/ (accessed Apr. 22, 2025).

58 The Foundation for Individual Rights and Expression (FIRE), Palsgaard v. Christian—Verified Complaint, https://www.thefire.org/research-learn/palsgaard-v-christian-verified-complaint (accessed Apr. 29, 2025).

59 The Foundation for Individual Rights and Expression (FIRE), After FIRE Lawsuit, California Community Colleges Will Not Enforce DEI Mandate in Classroom, (Feb. 10, 2025), https://www.thefire.org/news/after-fire-lawsuit-california-community-colleges-will-not-enforce-dei-mandate-classroom (accessed Apr. 29, 2025).

60 Feingold, *supra* note 39, at 250.

61
*Id*.

62 David Blumenthal et al., *Mirror, Mirror 2024: A Portrait of the Failing U.S. Health System*, Sept. 19, 2024, The Commonwealth Fund, https://www.commonwealthfund.org/publications/fund-reports/2024/sep/mirror-mirror-2024 (accessed Mar. 11, 2026).

63
*Id*.

64 Virginia Tangel et al., *Racial and Ethnic Disparities in Maternal Outcomes and the Disadvantage of Peripartum Black Women: A Multistate Analysis*, 2007–2014, 36 Am. J. Perinatology 835, 835, 843 (2019), https://www.thieme-connect.com/products/ejournals/html/10.1055/s-0038-1675207 (accessed Mar. 11, 2026).

65 Shannon Schumacher et al., *Five Facts About Black Women’s Experiences in Health Care*, KFF, May, 7, 2024, https://www.kff.org/racial-equity-and-health-policy/issue-brief/five-facts-about-black-womens-experiences-in-health-care/.

66 Kelly M. Hoffman et al., *Racial Bias in Pain Assessment and Treatment Recommendations, and False Beliefs about Biological Differences Between Blacks and Whites*, 113 Proc. Nat’l Acad. Sciences. No. 16, 4296, 4298–4301, Apr. 4, 2016, https://www.pnas.org/doi/10.1073/pnas.1516047113 (accessed Mar. 11, 2026).

67 Brad N. Greenwood et al., *Physician-Patient Racial Concordance and Disparities in Birthing Mortality for Newborns*, 117 Proc. Nat’l Acad. Sciences. No. 35, 21194, at 21194–21200 Aug. 17, 2020, https://www.pnas.org/doi/10.1073/pnas.1913405117?url_ver=Z39.88-2003&rfr_id=ori:rid:crossref.org&rfr_dat=cr_pub%20%200pubmed (accessed Mar. 11, 2026).

68 Jillian Olsen et al., *Racial Disparities in Hospital Mortality among Pediatric Cardiomyopathy and Myocarditis Patient*, 42 Pediatric Cardiology 59, 68, Oct. 6, 2020.

69 Jay Bhatt et al., *US Health Care Can’t Afford Health Inequities*, Deloitte Insights, June 22, 2022, https://www2.deloitte.com/us/en/insights/industry/health-care/economic-cost-of-health-disparities.html (accessed Apr. 30, 2025).

70 John E. Snyder et al., *Black Representation in the Primary Care Physician Workforce and its Association with Population Life Expectancy and Mortality Rates in the US*, JAMA, Apr 14, 2023, https://jamanetwork.com/journals/jamanetworkopen/fullarticle/2803898?resultClick=1.

71 Steven D. Pearson & Lisa H. Raeke, *Patients’ Trust in Physicians: Many Theories, Few Measures, and Little Data*, 15 J Gen Intern Med 509 (2000).

72 Lakshmi Nair & Oluwaseun A Adetayo, *Cultural Competence and Ethnic Diversity in Healthcare*, Plast Reconstr Surg Glob Open, May 16, 2019, DOI: http://dx.doi.org/10.1097/GOX.0000000000002219.

73
*U.S. Physician Workforce Data Dashboard*, AAMC, https://www.aamc.org/data-reports/report/us-physician-workforce-data-dashboard (accessed Mar. 7, 2025).

74
*New AAMC Data on Medical School Applicants and Enrollment in 2024*, AAMC, https://www.aamc.org/news/press-releases/new-aamc-data-medical-school-applicants-and-enrollment-2024 (accessed Apr. 23, 2025).

75 Georgetown University McCourt School of Public Policy, *Health Policy Institute, Cultural Competence in Health Care: Is it Important for People with Chronic Conditions?*, https://hpi.georgetown.edu/cultural/ (accessed Mar. 20, 2025).

76 LCME, *Standards for Accreditation of Medical Education Programs Leading to the MD Degree*, Standard 7.6, https://journalofethics.ama-assn.org/article/role-empathy-medicine-medical-students-perspective/2007-06 (accessed Mar. 11, 2026).

77 Minnesota Department of Management and Budget, *Enterprise Guidance on Fostering Diversity, Equity, Inclusion, and Cultural Competence in the Selection Process*, https://mn.gov/mmb-stat/hr-toolbox/006-recruit-and-retain/cultural-competency/dei-processes-in-selection-guidance-doc.pdf (accessed Mar. 11, 2026).

78 Soucek, *supra* note 26.

